# The Patient’s Physiological Status at the Start Determines the Success of the Inpatient Cardiovascular Rehabilitation Program

**DOI:** 10.3390/jcm12051735

**Published:** 2023-02-21

**Authors:** Anna Odrovicsné-Tóth, Bettina Thauerer, Barbara Stritzinger, Werner Kullich, Andreas Salzer, Martin Skoumal, Bibiane Steinecker-Frohnwieser

**Affiliations:** 1 Ludwig Boltzmann Institute for Arthritis and Rehabilitation, 5760 Saalfelden, Austria; 2 Rehabilitation Center of the Austrian Pension Insurance Company (PVA), 5760 Saalfelden, Austria; 3 Rehabilitation Center of the Austrian Pension Insurance Company (PVA), 1021 Vienna, Austria; 4 Ludwig Boltzmann Institute for Arthritis and Rehabilitation, 8962 Gröbming, Austria

**Keywords:** cardiovascular diseases, rehabilitation, advanced glycation end products, receptor for advanced glycation end products, AGE activity, CVD risk factors

## Abstract

Multidisciplinary inpatient rehabilitation plays an important role in the recovery of patients with cardiovascular diseases (CVDs). Lifestyle changes, achieved by exercise, diet, weight loss and patient education programs, are the first steps to a healthier life. Advanced glycation end products (AGEs) and their receptor (RAGE) are known to be involved in CVDs. Clarification on whether initial AGE levels can influence the rehabilitation outcome is important. Serum samples were collected at the beginning and end of the inpatient rehabilitation stay and analyzed for parameters: lipid metabolism, glucose status, oxidative stress, inflammation and AGE/RAGE-axis. As result, a 5% increase in the soluble isoform RAGE (sRAGE) (T_0_: 891.82 ± 44.97 pg/mL, T_1_: 937.17 ± 43.29 pg/mL) accompanied by a 7% decrease in AGEs (T_0_: 10.93 ± 0.65 µg/mL, T_1_: 10.21 ± 0.61 µg/mL) was shown. Depending on the initial AGE level, a significant reduction of 12.2% of the AGE activity (quotient AGE/sRAGE) was observed. We found that almost all measured factors improved. Summarizing, CVD-specific multidisciplinary rehabilitation positively influences disease-associated parameters, and thus provides an optimal starting point for subsequent disease-modifying lifestyle changes. Considering our observations, the initial physiological situations of patients at the beginning of their rehabilitation stay seem to play a decisive role regarding the assessment of rehabilitation success.

## 1. Introduction

Although the global mortality rate for cardiovascular diseases (CVDs) has declined in recent years, CVDs remain the leading cause of death in the Western population. The inflammatory processes of atherosclerosis, a disease that functions as an inflammatory systemic disease affecting the arterial walls, are involved in all stages of atherosclerosis formation [1]. Efforts in the prevention of CVDs and their associated risk factors are required to mitigate the epidemic.

Advanced glycation end products (AGEs) have been previously discussed and described in the context of cardiological diseases [2]. AGEs, usually associated with aging and diabetes, are modified molecular products, produced by nonenzymatic reactions between the aldehydic group of reducing sugars with proteins, lipids or nucleic acids [3]. They are formed especially in hyperglycemia; however, the production of AGEs also occurs in processes characterized by oxidative stress and inflammation, such as in the development of atherosclerosis. AGEs can also be incorporated in the forms of food that have been exposed to high temperatures, such as through frying, grilling or toasting, and sugar-rich foods. AGEs contribute to vascular damage and the occurrence of atherosclerotic plaque progression through the alteration of the functional properties of molecules of the extracellular matrix of vessel walls, or through activation of cell receptor-dependent signaling [4]. AGE effects are triggered by their binding to their specific receptors (receptor for advanced glycation end products, RAGE). This transmembrane signaling receptor is present in all cells associated with atherosclerosis and is able to influence cellular function, promote gene expression, and to enhance and release proinflammatory molecules. The AGEs can increase the production of reactive oxygen species (ROS) and initiate intracellular oxidative stress. Conversely, the increase in ROS production can in turn promote the production of AGEs, thereby forming a vicious circle between oxidative stress and AGEs [4]. The soluble form of the receptor for AGEs (sRAGE), however, is known to bind circulating AGEs and plays therefore a competitive role. Because the transmembrane domain is lacking, no signal can be forwarded. Thus, a high protective effect can be attributed to the soluble receptor, indicating that the ratio between AGEs and sRAGE acts as an important factor and seems to be more meaningful than the individual values. Because of this reason, we declared the quotient of AGE/sRAGE as AGE activity, which can also serve as an universal biomarker for various diseases [5,6,7,8,9,10].

AGEs bind and accumulate to collagen and elastin in the epithelium and dermis [11]. Several studies have demonstrated that skin autofluorescence reflects accumulation of AGEs in the layers of the skin and tissue levels, because they emit light when exposed to ultraviolet waves [3,12]. Moreover, it has been revealed that AGE measured in skin biopsies correlate with the measurements of skin autofluorescence performed with an AGE reader [13].

One of the proinflammatory enzymes involved in the process of atherosclerosis is myeloperoxidase (MPO). MPO is derived from granules of activated neutrophil granulocytes, monocytes and macrophages and enhances the oxidative potential of its co-substrate hydrogen peroxide by formation of potent oxidants [14,15,16]. The study of Kataoka et al. showed a greater progression of atherosclerosis in diabetic patients by the elevated systemic MPO levels [17]. In addition to findings that MPO promotes endothelial dysfunction and enhances atherosclerotic plaque formation, myocardial infarction also occurs via MPO [18,19]. These observations also underpin the development of therapeutic interventions targeting MPO.

Today, adipose tissue is considered as an endocrine organ that releases biologically active factors called adipokines, such as the retinol binding protein-4 (RBP-4) [20,21]. Especially in women, RBP-4 is considered as an established risk factor and is presumed as a predictor for CVDs [22]. Huang et al. demonstrated that patients with higher RBP-4 levels experience an enormous risk of coronary artery calcification [23]. The results of a study over a 10-year follow-up period showed that elevated RBP-4 levels in childhood may predict cardiometabolic risk in adulthood [24]. In serum, RBP-4 levels are negatively correlated with high-density lipoprotein (HDL) but positively correlated with LDL-cholesterol, total cholesterol and triglyceride, indicating its role in lipid metabolism [25].

Therefore, a cardiac rehabilitation program with a balanced diet could aim, among other things, to reduce the levels of lipid metabolic parameters such as cholesterol, low density lipoprotein (LDL), high density lipoprotein (HDL) and triglycerides (TGL) [26,27].

The prevalence of CVDs is rapidly increasing in the world and CVD has been positively linked with diabetes mellitus (DM), particularly type 2 diabetes mellitus (T2DM) [28]. DM is a group of metabolic diseases characterized by hyperglycemia, resulting from defects in insulin secretion or response to insulin [29]. Several studies have demonstrated that the elevated levels of long-term glucose, verifiable via Hemoglobin A1c (HbA1c), might contribute to the development of CVDs and are associated with increased risk of death. It was revealed that changes in lifestyle (e.g., quitting smoking, reducing alcohol consumption, regular physical activity, healthy and optimal glycemic control) can lead to an improvement in CV risk and prevention of CVD events [30,31,32,33,34,35].

The most widely recognized marker of inflammation is the C-reactive protein (CRP), synthesized primarily by the liver [36]. Koenig et al. showed that it is released in response to acute inflammatory stimuli and is considered a risk biomarker for cardiovascular events [37]. CRP plays an important role in the atherosclerotic process and acts at early and key stages of plaque formation. Caulin-Glaser et al. showed an improvement in CRP levels in patients who participated in a cardiac rehabilitation program [38].

Evaluation of cardiac performance is often based on the result of ergometer training and maximum exercise capacity, which is equivalent to reaching a theoretical maximum heart rate [39]. In the present study, maximal cycle ergometry cardiovascular responses of individuals participating in a cardiac inpatient rehabilitation program via ergometric parameters (Watt value as well as the age- and weight-related expected cardiovascular exercise capacity) between the beginning and end of the rehabilitation stay were measured.

One of the risk factors contributing to the CVDs is hypertension, as it is a significant contributor to CVD-related morbidity and mortality [40]. Normal blood pressure for adults is defined as a systolic pressure of less than 120 mmHg and a diastolic pressure of less than 80 mmHg, while readings of more than 130–139/80–89 mmHg signify hypertension [41]. A cornerstone of hypertension treatment is lifestyle modification and increased exercise and smoking cessation, among other things, which are encouraged in cardiac rehabilitation; in addition, the introduction of a Mediterranean diet after rehabilitation can be helpful [42].

Obesity, as well as higher body mass index (BMI), has consistently been associated with an increased risk for metabolic diseases and CVDs [43]. Being overweight can directly increase the risk of restrictive cardiomyopathy and heart failure due to diastolic dysfunction [44].

On the basis of these data, we aimed to demonstrate whether crucial CVD risk factors can be positively influenced by inpatient cardiac rehabilitation and whether the patient’s baseline condition may affect the success of rehabilitation.

## 2. Materials and Methods

This study was conducted as a cross-sectional study at the rehabilitation center of the Austrian pension insurance company (PVA) in Saalfelden, from 05/2016 to 04/2017. The procedures described were in accordance with the ethical standards of the Ethics Committee of Salzburg (415-E/1988/7-2016) as well as with the Helsinki Declaration. The medical superintendent of the PVA approved the concept. The study was recorded in the German Clinical Trials Register (DRKS00010509). 

### 2.1. Study Design

The data and serum of the investigated subjects were collected at admission (baseline, T_0_) and release (discharge, T_1_) following 3–4 weeks of inpatient rehabilitation stay. The cardiac therapy-induced changes were analyzed by pre–post analysis, comparing T_0_ with T_1_. Inclusion criteria were presence of a coronary heart disease such as status post myocardial infarction, PTCA/stent implantation, coronary artery bypass graft surgery, and an age limit of 25–75 years. Exclusion criteria were chronic kidney disease from stadium III (glomerular filtration rate ≤ 60 mL/min), acute inflammatory disease (CRP cut off ˃ 1.0 mg/dL), operation or greater trauma < 6 weeks ago, myocardial infarction < 4 months, pregnancy or lactation period, as well as alcohol and drug abuse. The selection of patients and admission to the study was conducted by doctors of the rehabilitation center Saalfelden. All patients agreed to participate after verbal and written informed consent. Their data were processed anonymously, and data protection was observed in the current version.

### 2.2. Patients Collective

Sixty-six patients of the rehabilitation center Saalfelden, of the PVA, were included in this study. Seven of them dropped out: two because of termination of the rehabilitation program, one missed the appointment for skin autofluorescence measurement, one was older than 75 (one of the inclusion criteria) and three of them had higher CRP values (˃1.0 mg/mL). Patients in this study attended rehabilitation with the main indication of cardiovascular disease, and mainly exhibited a decrease in performance, dyspnea, and thoracic discomfort. Therefore, the data of 59 patients (45 men, 14 women) in the age range of 33–75 years with coronary heart disease were used for further investigation. All patients underwent an inpatient multidisciplinary rehabilitation program for 3–4 weeks with active and passive physical therapy. This program comprised a comprehensive rehabilitation medical admission process, the creation of an individual training program, exercise therapy with strength training (this is tailored to the patient and to prevent overload, each workout is controlled by heart rate monitoring), indication-specific training courses, diet and nutritional counseling, patient education program as well as psychosocial support.

### 2.3. Investigations of Serum Samples via Routine Laboratory and ELISA Measurements

The following laboratory parameters were taken from the routine laboratory examination at the Saalfelden Rehabilitation Center: fasting blood glucose, CRP, total cholesterol, LDL, HDL, TGL as well as HbA1c. Serum blood samples were collected at baseline and discharge, centrifuged within an hour, and stored at −80 °C. The measurement of special parameters MPO and RBP-4 was conducted by commercial enzyme linked immunosorbent assay (human myeloperoxidase-ELISA, Hölzel Diagnostika Abfrontier, Cat. No. LF-EK0134; retinol binding protein-ELISA, Immundiagnostik, Cat. No. KG6110). In addition, AGE and sRAGE levels were measured by ELISA technique (OxiSelect Advanced Glycation End Product (AGE) Competitive ELISA Kit, Cat. No. STA-817; human sRAGE ELISA, Cat. No. RD191116200R). ELISA measurements were conducted in duplicate to ensure quality.

For a better and more understandable representation of the AGE ligand–receptor system, AGE activity was formed from the AGE/sRAGE ratio. A decrease in AGE activity describes rehabilitation success by representing the decrease in AGEs versus the increase in protective sRAGE.

### 2.4. Statistical Analysis

All statistical analysis was performed using the statistic program GraphPad Prism 9. Pairwise deletion was used for completely randomly distributed missing data. Normal distribution was assessed using the Shapiro–Wilk test. If data were normally distributed, pre–post groups were compared using paired Student´s t-test, otherwise Wilcoxon matched pairs signed rank test was applied. Correlations were assessed using the Spearman´s rank correlation coefficient (r). Results are expressed as mean ± standard error of mean (SEM) or individual values plus mean.

In order to analyze the effects of prior physical activity on specific parameters, the patients were divided into two groups based on their daily activities before the rehabilitation (sport+: patients engaged in 30 min sport activity daily, sport−: patients did not engage in 30 min sport activity daily).

### 2.5. Skin Autofluorescence (SAF) Measurement

SAF was measured from the inside of the dominant forearm using an AGE reader (DiagnOptics Technologies BV, Groningen, Netherlands) in a non-invasive way. The AGE reader illuminates skin with ultraviolet light and detects the resulting fluorescent light, while simultaneously detecting light reflected from the skin. SAF is determined as the ratio of light intensity in the 420–600 nm wavelength range and the average excitation light intensity in the 300–420 nm range.

### 2.6. Physical Performance

Individual physical performance was documented at baseline (T_0_) and discharge (T_1_) with the Ergoselect 200 Ergometer (Ergoline GmbH) by the AMEDTEC ECGpro software, according to the manufacturer’s instructions.

## 3. Results

### 3.1. Demographic Data

At the beginning of the rehabilitation program, data concerning the frequency distribution of the diagnosis from included patients, with a mean age of 58 (±1.11; 76% (n = 45) males, 24% (n = 14) females), were collected (Table 1). Myocardial infarction (MI) and percutaneous coronary intervention (PTCA) showed the highest number of patients, followed by diabetes, coronary artery bypass graft (CABG), positive ergometry and others (e.g., peripheral arterial disease, biodegradable stent, Hashimoto-thyroiditis, arterial hypertension, chronic wound healing, drug eluting stents, positive family history for coronary heart disease, positive risk profile). Most patients were taking nonsteroidal anti-inflammatory drug(s) (NSAIDs) (e.g., Aspirin), statins or ß-blockers. A smaller amount took calcium channel blockers and nitrate derivatives. With regard to medication, no significant changes were observed when baseline values were compared with those at discharge.

### 3.2. Physiological Improvements

**Figure 1:**
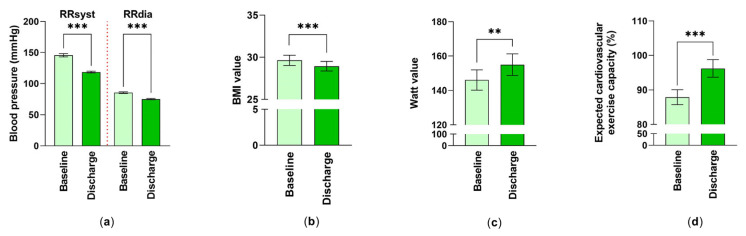
The effect of inpatient cardiac rehabilitation on physiological parameters: (**a**) Differences in systolic and diastolic blood pressure are shown. Mean ± standard error of mean (SEM) are given. ***: *p* < 0.001 (n_syst, dia_ = 59); (**b**) Changes in BMI values were examined. Values were expressed as mean ± SEM. ***: *p* < 0.001 (n = 59); (**c**,**d**) Values of cycle ergometry were studied. Measured Watt-values (n = 52) and expected cardiovascular exercise capacity (%) (n = 56) at baseline and at discharge expressed as averaged data ± SEM. Statistical analysis for these comparisons were performed. **: *p* < 0.01, ***: *p* < 0.001.

In this study, physiological parameters such as blood pressure, body weight expressed as BMI, and physical performance, were collected. A statistically significant improvement was observed for both RR–systolic (18.54% reduction) and RR–diastolic (12.21% reduction) by the inpatient rehabilitation stay (Figure 1a).

The examined rehabilitation patients with cardiovascular diseases were overweight with a body mass index of 29.63 ± 0.60 (mean ± SEM). After the 3–4-week rehabilitation stay, patients, although still in the overweight range, showed significantly decreased BMI values of 28.94 ± 0.56 (mean ± SEM) at discharge (Figure 1b). Accordingly, the weight and abdominal girth of the examined rehabilitation patients improved during the rehabilitation (weight at T_0_: 87.92 ± 2.27 kg, weight at T_1_: 85.77 ± 2.08 kg (mean ± SEM, *p* < 0.001, n = 59) girth at T_0_: 104.60 ± 1.64 cm, girth at T_1_: 102.7 ± 1.67 cm (mean ± SEM, *p* < 0.001, n = 53)).

Values for the bicycle ergometer training were improved over the 3-4-week rehabilitation period regarding wattage, from 146.1 ± 5.91 at baseline to 155.0 ± 6.28 (mean ± SEM) (6.09% improvement) at discharge and in terms of the expected cardiovascular exercise capacity, from 87.89 ± 2.17% to 96.21 ± 2.53% (mean ± SEM) (9.47% improvement) (Figure 1c,d).

### 3.3. AGEs and sRAGE in CVD Patients

Tissue accumulation of AGEs in the skin was measured non-invasively via the AGE Reader. Because AGEs accumulate in tissues (e.g., skin) over a person’s lifetime, it is not surprising that the 3–4 weeks of the inpatient rehabilitation stay could not change these values significantly (at baseline: 2.39 ± 0.06 AU, at discharge: 2.43 ± 0.06 AU (mean ± SEM)).

**Figure 2:**
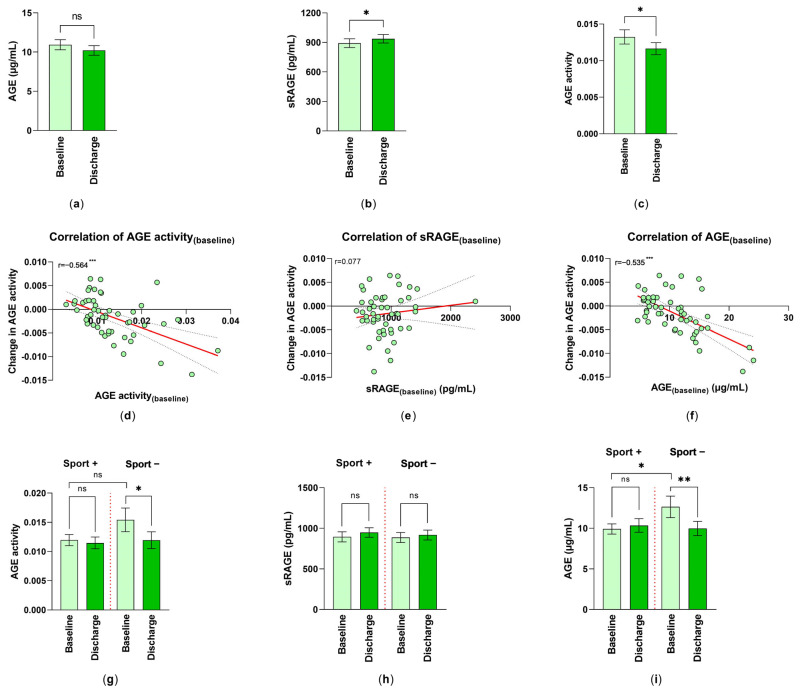
Changes in AGE ligand–receptor system by inpatient rehabilitation: (**a**) AGE levels (n = 51); (**b**) sRAGE (n = 55); and (**c**) AGE activity (AGE/sRAGE-quotient) (n = 51) are shown as mean ± SEM. Statistical differences between baseline (T_0_) and discharge (T_1_) were determined via the Wilcoxon matched pairs signed rank; *: *p* < 0.05. Spearman correlation (r) of AGE activity_(changes)_ (T_1−_T_0_) with (**d**) AGE activity_(baseline)_, (**e**) sRAGE_(baseline)_ and (**f**) AGE_(baseline)_, respectively, were analyzed (n = 51); simple linear regression is given (red line); statistical relevance for (**d**,**f**): ***: *p* < 0.001. The analysis of the effect of physical activity on (**g**) the changes of AGE activity (n_Sport+_ = 32, n_Sport−_ = 19); (**h**) the changes in the serum levels of sRAGE (pg/mL) (n_Sport+_ = 35, n_Sport−_ = 20); and (**i**) the serum levels of AGE (n_Sport+_ = 32, n_Sport−_ = 19) were performed. Differences between the patient groups with/without daily exercises were measured and the values were expressed as mean ± SEM. *: *p* < 0.05, **: *p* < 0.01.

However, measurements of AGEs in serum showed a nearly 7% reduction in the AGE levels from 10.93 ± 0.65 µg/mL at T_0_ to 10.21 ± 0.61 µg/mL (mean ± SEM) at T_1_ (Figure 2a).

By comparing the concentration of sRAGE at baseline and discharge, an increase from 891.82 ± 44.97 pg/mL to 937.17 ± 43.29 pg/mL (mean ± SEM) could be observed (Figure 2b).

### 3.4. Changes in AGE Activity and the Rehabilitation

The AGE activity was formed out of the quotient AGE/sRAGE. The AGE activity showed a significant reduction of approximately 12.15%, which can be equated with the rehabilitation success of the included patients (Figure 2c). By further focusing on AGE activity, their AGE activity_(changes)_ (change = AGE activity_(discharge)_–AGE activity_(baseline)_) was correlated to AGE activity_(baseline)_. There was a significant mediate negative correlation (r = −0.564), showing that the higher the AGE activity at the start of rehabilitation, the bigger the AGE activity_(changes)_ (T_0_ versus T_1_), resulting in a more negative value (Figure 2d). The correlation between AGE activity_(changes)_ and sRAGE_(baseline)_ was not pronounced (Figure 2e). On the other hand, the correlation between the AGE activity_(changes)_ and AGE_(baseline)_ was highly significant (r =−0.535) (Figure 2f). Despite this, a significant correlation between AGE activity_(baseline)_ and AGE activity_(discharge)_ (r = 0.653; *p* < 0.001) was calculated.

### 3.5. The Effect of Preceding Physical Activity on the Success of Rehabilitation

Physical fitness can modify the success of the rehabilitation, and therefore patients were asked for their self-reported daily exercises, conducted prior to rehabilitation. About 66% of the subjects indicated that they performed 30 min of physical activities per day. Because of this reason, we investigated the changes in AGE activity at baseline (T_0_) and discharge (T_1_) in patients with/without prior physical activities. It was obvious that non-sporty-active patients already had higher AGE activity levels at the beginning of the rehabilitation than sporty-active patients; however, due to the small evaluable number, this was not significant. AGE activity decreased in the no-sport group significantly following the rehabilitative measures (Figure 2g). A comparison of the serum levels of sRAGE did not show a significant improvement at discharge (Figure 2h). It is of great interest that AGE per se was higher in the no exercise group. Inpatient rehabilitation significantly reduced AGE. Because of the already lower AGE level in the sport group, no improvement could be detected (Figure 2i).

### 3.6. Analysis of Risk Factors of Cardiovascular Diseases

**Figure 3:**
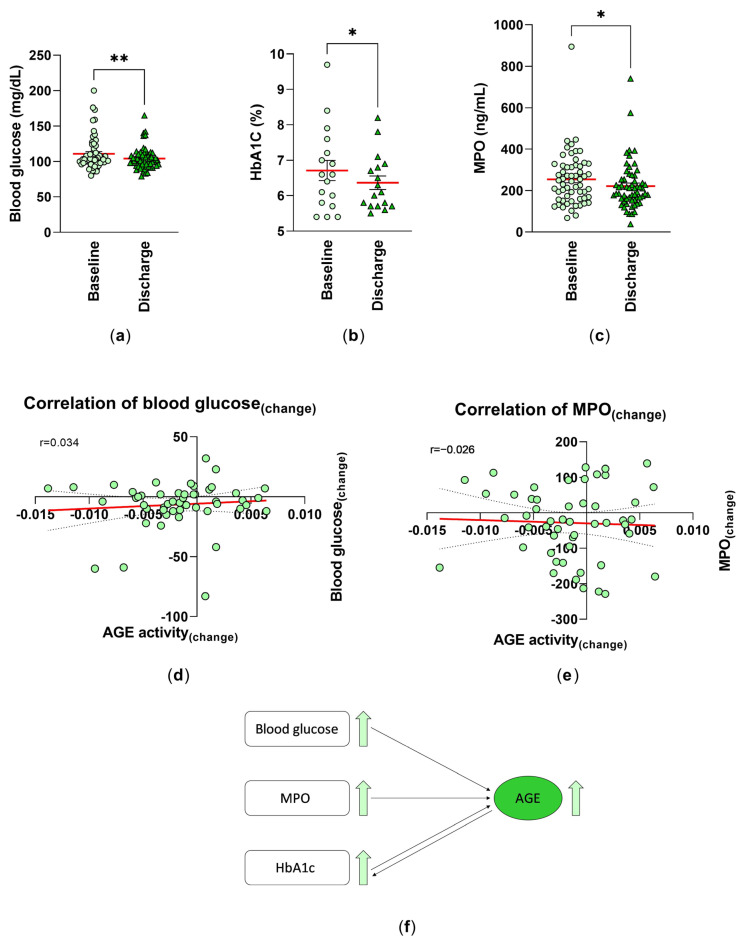
Analysis of the risk factors for cardiovascular diseases: the blood levels of (**a**) glucose (mg/dL) (n = 58), (**b**) HbA1c (%) (n = 17) were measured as part of the routine blood analysis, and (**c**) the MPO levels of serum samples (ng/mL) (n = 59) were analyzed by ELISA technique at baseline (T_0_) and discharge (T_1_). Individual and mean values (red line) are shown. **: *p* < 0.01; *: *p* < 0.05. Spearman correlation (r) between the AGE activity_(changes)_ and (**d**) blood glucose_(changes)_ (n = 50) and (**e**) the MPO_(changes)_ (T_1−_T_0_) of the serum samples (n = 51) were analyzed. Simple linear regression is given (red line). (**f**) Schematic representation of the relationship between the CVD risk factors and serum levels of AGEs.

Serum glucose concentrations are a continuous risk factor for CVDs. Blood glucose levels in patients included in our study decreased significantly from 110.7 ± 3.10 mg/dL at baseline to 104.2 ± 2.02 mg/dL (mean ± SEM) at discharge (Figure 3a). Several studies have demonstrated that a higher HbA1c level is also associated with an increased risk of cardiovascular diseases and death. In this study, we were able to investigate the impact of the inpatient rehabilitation on HbA1c serum level by showing a significant decrease from 6.71 ± 0.29 (%) at baseline to 6.36 ± 0.19 (%) at discharge (Figure 3b). Although the HbA1c value indicates changes in blood glucose levels over a period of 3–4 months, we were able to detect a significant difference after only 3–4 weeks. However, despite this significance, this is only an indication of improvement, as only n = 17 patients were studied here, which does not allow for good reliability. This decrease was also accompanied by an improvement in MPO from 254.1 ± 16.68 ng/mL to 221.3 ± 14.94 ng/mL (Figure 3c). A possible correlation between the AGE activity_(changes)_ and the blood glucose_(changes)_ or MPO_(changes)_ was proven. A positive relationship (r = 0.034)—meaning that the higher the changes in AGE activity, the higher the changes in blood glucose level—was detected (Figure 3d). Whereas, a negative correlation was shown for MPO (r =−0.02) (Figure 3e).

We can schematically summarize our collected data and show the relationship between CVD risk factors and serum levels of AGEs. Higher blood glucose and MPO levels may lead to higher AGE concentration. HbA1c is a result of advanced glycation, but can also act as a precursor for Hb-AGE, contributing to higher AGE levels (Figure 3f).

### 3.7. Lipid Metabolism, RBP-4 and Rehabilitation

**Figure 4:**
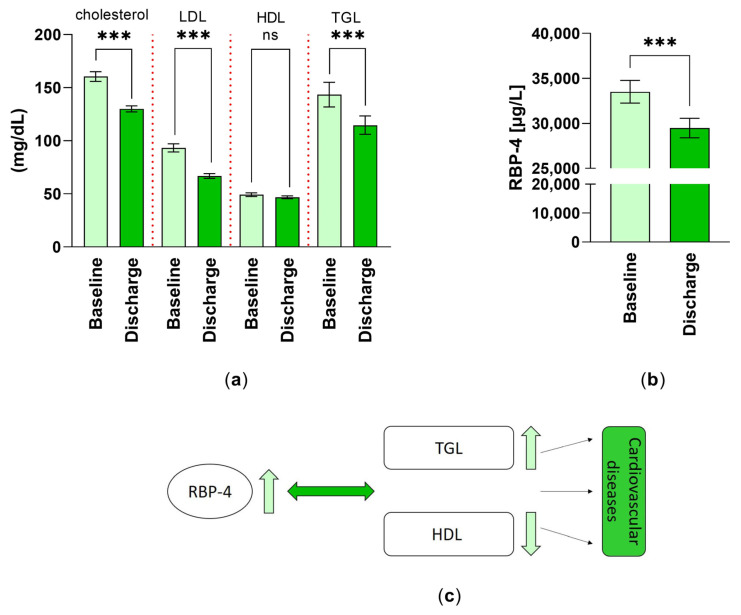
Changes in factors of lipid metabolism: (**a**) the cholesterol, low density lipoprotein (LDL), high density lipoprotein (HDL) and triglyceride (TGL) levels (mg/dL) (n = 59) were analyzed as part of the routine blood analysis and are expressed as mean ± SEM. ***: *p* < 0.001; (**b**) differences in retinol-binding protein-4 (RBP-4) levels (µg/L) (n = 55) were measured at T_0_ and T_1_ by ELISA technique and are expressed as mean ± SEM. ***: *p* < 0.001; (**c**) summary of the effect of lipid metabolism (TGL, HDL) and serum levels of RBP-4 on cardiovascular diseases.

Important parameters for the lipid metabolism were investigated (Figure 4a). The cholesterol levels of the patients significantly decreased by 19% at discharge (T_1_). At discharge (T_1_), the patients showed a 28.55% decline in LDL level, a 4.92% reduction in HDL level, and a 20.01% reduction in TGL level.

In the course of the 3–4 weeks of inpatient rehabilitation, the mean serum level of the parameter RBP-4 decreased significantly by 12.01% (Figure 4b).

The cartoon in Figure 4c summarizes the relationship between the heightened level of RBP-4 and the increase in lipid metabolism, by a reduction in the good cholesterol HDL and an increase in triglycerides. Respectively, the further effects of those on cardiovascular diseases are depicted.

## 4. Discussion

Several studies have shown that AGEs play a crucial role in the aging process as well as the development of tumor metastasis, and they can contribute to the development of CVDs [45]. Falcone et al. (2005) demonstrated the inverse correlation between the levels of sRAGE and the degree of atherosclerosis [46]. Ebert (2019) and her group reported that the ratio of AGEs and sRAGE is a more important marker for age-related diseases than each separately [47]. Confirming this work, we formed the quotient of AGE/sRAGE and named this as AGE activity. Our results proofed the finding of a previous study, which demonstrated that changes in lifestyle can lead to an increase in sRAGE serum levels as well as a decrease in AGE levels and AGE activity [48]. Furthermore, our findings were in line with Hangai et al., who described that the skin autofluorescence measurement using the forearm did not match with serum AGEs [49].

Interestingly, our results showed that AGE serum concentration measured at the beginning of rehabilitation (admission/baseline) seems to be very important for rehabilitation outcome, in the context of applying a CVD-specific rehabilitation program. Physical exercise is one of the most effective non-pharmacological treatments to reduce the risk of atherosclerosis and cardiovascular diseases. It has positive effects on the vascular system. Consistent with these studies, our data also clarified the association between physical activity and lower AGE levels, meaning that patients with no exercise activity had higher AGE levels at admission than subjects who exercised daily [50]. Interestingly, we were able to show that in patients who did not exercise and had a higher baseline level of AGE, these levels were significantly reduced at the end of rehabilitation. Earlier findings of various research groups [51,52] demonstrated that the glycation process in vivo results in two different products, the early reversible products (Schiff bases and Amadori adducts) and the advanced glycation end products (AGEs). Glycated hemoglobin (HbA1c), as a result of early glycation, is a precursor for Hb-AGE formation by slow and complex rearrangements. In addition, serum AGE levels were significantly higher in patients with higher HbA1c levels [53]. Studies report a positive relationship between the elevated levels of blood glucose, HbA1c, as well as MPO and the higher levels of AGEs [54,55,56]. Our findings showed an improvement in these parameters after the 3-4-week intensive rehabilitation program. From this, we can also infer that the observed reduction in AGE activity could be regulated by the rehabilitative effect on the above-mentioned parameters’ observations, once again highlighting the effectiveness of the applied rehabilitation process in patients with CVD.

On the contrary, we could not show a significant decrease in the CRP level after the 3–4-week rehabilitation stay, being possibly related to the limited CRP value at baseline.

The effective rehabilitation exercise program and the dietary change instructed by a dietician may be responsible for the constant improvement in all parameters of lipid metabolism (total cholesterol, LDL, HDL and triglycerides) during the inpatient rehabilitation stay.

RBP-4 is considered as an important risk factor for plaque formation in carotids and the development of cardiovascular diseases [57]. A study of Liu Y et al. indicates a context between the severity of coronary complex lesions and RBP-4. This study, conducted among more than 600 patients, shows that RBP-4 is suitable as a predictor for cardiac death [58]. Zabetian-Targhi et al. published that there is a positive relationship between RBP-4 and oxidative stress marker [59]. High RBP-4 increases blood pressure, and absence of RBP-4 decreases blood pressure, meaning that accelerated RBP-4 levels are correlated with the prevalence of hypertonia and myocardial infarction [60]. In patients with movement training (3 days/week) over a period of 12 months, monitored by physical therapists, decreased RBP-4 levels are accompanied by lower triglyceride levels [61]. These results correlate with our findings which show, already, a decrease in RBP-4 after 3–4 weeks of rehabilitation treatment, and indeed the patients underwent dietary measures. A meta-analysis has also demonstrated that cardiac rehabilitation participation is associated with reductions in blood pressure [62]. In addition, the BMI and blood pressure values improved in the rehabilitation patients, in the form of a reduction in the course of rehabilitation, while a mild correlation between BMI and blood pressure was detected.

## 5. Conclusions

Our results impressively show that a 3–4-week inpatient rehabilitation stay related to CVD has a positive effect on physiological parameters, serum levels of AGEs, and sRAGE and AGE activity. Furthermore, the specific rehabilitation program resulted in an improvement in cardiovascular risk factors and lipid metabolism, body mass index, and serum levels of RBP-4 in subjects with CVDs. Moreover, we were able to justify the fact that the investigation of cardiovascular risk predictors plays a crucial role in the cardiac rehabilitation outcomes.

## Figures and Tables

**Table 1:** Summary of the demographic data. Gender distribution, mean age of the patients (mean ± SEM), distribution of diagnosis and medication at baseline (T_0_) are given. Some patients had multiple diagnoses. Myocardial infarction (Status post MI), percutaneous coronary intervention (St.p. PTCA), coronary artery bypass graft (St.p.CABG), positive ergometry (pos.Ergo) and nonsteroidal anti-inflammatory drugs (NSAIDs).

Demographic Data			
**Gender distribution of the patients**			
	**n**	**%**	
male	45	76%	
female	14	24%	
all	59	100%	
**Mean age of the patients**			
male	57.7 ± 1.13		
female	59.1 ± 3.03		
all	58.0 ± 1.11		
**Distribution of the diagnoses of the patients**			
	**male (n)**	**female (n)**	**all (n)**
St.p.MI	30	10	40
St.p.PTCA	42	14	56
St.p.CABG	3	0	3
pos.Ergo	1	2	3
Others	3	2	5
**Medication of the patients**			
	*Baseline*		
	**male (n)**	**female (n)**	**all (n)**
NSAIDs Aspirin	40	13	53
Statins	44	12	56
ß-blockers	35	10	45
Calcium channel blockers	2	2	4
Nitrate derivatives	2	0	2

## Data Availability

Restrictions apply to the availability of these data. Data was obtained from Austrian Pension Insurance Company (PVA) and are available with the permission of PVA.
